# Trends and Regional Differences in Usage of Primary External Fixation From 2018 to 2022 in Japan: A Retrospective Observational Study Using Open Data from the National Database of Health Insurance Claims

**DOI:** 10.7759/cureus.79854

**Published:** 2025-02-28

**Authors:** Natsumi Saka, Kentaro Matsui, Yoshinobu Watanabe, Hirotaka Kawano

**Affiliations:** 1 Orthopaedic Surgery, Teikyo University School of Medicine, Tokyo, JPN

**Keywords:** comminuted fractures, damage control, insurance system, multiple trauma, open fractures, orthopaedic trauma

## Abstract

Background

Primary external fixation (EF) is a crucial method in orthopedic trauma, particularly for complex fractures with soft tissue damage. Despite its importance in damage control surgery, the patterns of EF use in Japan remain underexplored. This study aimed to elucidate the trends and regional differences in EF usage for fracture treatment in Japan and identify factors associated with its variation across regions.

Methods

We analyzed data from the National Database of Health Insurance Claims (NDB) covering inpatient surgeries from 2018 to 2022. The primary outcome was the EF usage ratio, calculated as the number of primary EFs procedures divided by the total number of internal fixations. The trend of the EF usage ratio over five-year period was assessed by Cochrane-Armitage trend test for temporal trends. We also evaluated the associations between EF usage and factors such as population, region, and emergency care availability.

Results

A total of 610,326 internal fixations and 29,546 EF procedures were identified, with the EF usage ratio increasing from 4,532 out of 119,223 cases (3.8%) in 2018 to 6,861 out of 126,000 cases (5.4%) in 2022 (p < 0.01). Regional EF usage in total of five-year period ranged from 228 out of 8,103 cases (2.8%) in Oita to 814 out of 9,075 cases (9.0%) in Nagasaki, representing a threefold difference. While 12 prefectures showed statistically significant increases in EF usage, no specific factors were found to explain the regional variations in EF utilization.

Conclusion

EF usage in Japan has risen over the past five years, with notable regional differences. Further research is needed to identify the causes of these variations and promote consistent trauma care across the country.

## Introduction

Primary external fixation (EF) is a critical strategy in trauma management, particularly in emergency settings, as a temporary stabilization tool for complex and open fractures [1-4]. It is also recommended in cases involving fractures with severe soft tissue damage, significant comminuted diaphyseal fractures, complex joint fractures, and serious ligamentous injuries [3,4]. The use of EF allows surgeons to wait definitive fracture treatment until soft tissue damage has stabilized or the patient's vital signs have been adequately stabilized [3]. Despite the recommendations in major orthopedic trauma guidelines, including those from the US, the utilization of EF for orthopedic trauma in Japan remains unclear [3].

 The Japanese National Database of Health Insurance Claims (NDB), managed by the Ministry of Health, Labour and Welfare, serves as a key tool for assessing the current use of medical devices. As one of the world's largest healthcare databases, it offers comprehensive insights into health services provided through Japan's universal healthcare system. The NDB data comprises electronic claims pertaining to outpatient visits, inpatient admissions, diagnosis and procedure combinations, prescriptions, dental treatments, and specific health checkups for each patient. Basic aggregate tables from the NDB are publicly available online as 'NDB Open Data.' These tables present statistics categorized by prefecture, gender, and age group [5-7]. NDB data have been utilized in orthopedic research, including studies on the epidemiology of hip fractures, meniscus surgery, and Achilles tendon rupture [6,8,9]. While primary EF has been utilized in Japan for decades in managing severe orthopedic trauma, specific reimbursement for this procedure under Japanese health insurance only began in 2018. Consequently, there are no prior published reports on EF usage in Japan.

Regional differences and trends in EF usage in Japan may indicate progress toward standardized orthopedic trauma care nationwide. Standardized orthopedic trauma care emphasizes delivering consistent, evidence-based treatments to patients with musculoskeletal injuries. Implementing standardized protocols, such as dedicated trauma care rooms and structured care pathways, has demonstrated improvements in patient outcomes, cost-effectiveness, and overall quality of care [10,11]. However, orthopedic trauma care in Japan lacks standardization, with significant variability in trauma care environments that may lead to the regional difference in EF usage. To establish the standardization of the orthopedic trauma care in Japan, there is a need to clarify the current use of primary EF. Using the NDB open data, this study aims to elucidate the temporal trends in the number of cases and the usage ratio of primary EF in the fracture treatment in Japan, as well as the regional differences in the ratio and the related factors. We hypothesized that the temporal trends in the number of cases and the usage ratio of primary EF is increasing due to the new reimbursement for EF.

## Materials and methods

Our work adhered to the REporting of studies Conducted using Observational Routinely-collected health Data (RECORD) Statement [12].

Data collection

We downloaded the inpatient surgical data (number of procedures categorized by prefecture and fiscal year) from 2018 to 2022 from the website of NDB open data [7]. Data collection was performed on Jun 30, 2024. From inpatient surgical data, we extracted the number of primary EFs (K046-3, code 150395810), the number of open reduction and internal fixation (ORIF) for fractures (K046) in the forearm (code 150019310)/lower leg (code 150019410), intra-articular fractures (K073) in knee (code 150042810)/hand (code 150043110)/ foot (code 150043210), and the number of internal fixations for pelvic fractures (K125, code 150060910). When the number of the surgeries is less than 10, the open NDB data reported the number as "-". In that case, we substituted "-" as zero. Patients who undergo primary EFs typically proceed to internal fixation afterward. Therefore, we estimated the EF usage ratio by dividing the number of primary EFs by the total number of internal fixations. The ratio was expressed as percentage. We did not include the number of femur fractures and humeral fractures as denominators, since the estimated ratio of primary EFs among those fractures are small and the result can be distorted by the large number of proximal femoral fractures in older adults and pediatric humeral supracondylar fractures, which generally do not require placement of primary EF.

To evaluate the association between EF usage ratio in each prefecture and related factors, we used the population of Japan stratified by prefecture, as reported by the Statistics Bureau of Japan [13]. The prefecture was consolidated into eight regions; 1. Hokkaido Region: Hokkaido; 2. Tohoku Region: Aomori, Iwate, Miyagi, Akita, Yamagata, Fukushima; 3. Kanto Region: Tokyo, Kanagawa, Chiba, Saitama, Ibaraki, Tochigi, Gunma; 4.Chubu Region: Niigata, Toyama, Ishikawa, Fukui, Yamanashi, Nagano, Gifu, Shizuoka, Aichi; 5. Kansai Region: Osaka, Kyoto, Hyogo, Nara, Shiga, Wakayama, Mie; 6. Chugoku Region: Okayama, Hiroshima, Yamaguchi, Shimane, Tottori; 7. Shikoku Region: Kagawa, Tokushima, Ehime, Kochi; 8. Kyushu Region: Fukuoka, Saga, Nagasaki, Kumamoto, Oita, Miyazaki, Kagoshima, Okinawa. The number of tertiary emergency and critical care centers per one million people in each prefecture was extracted from the data published from the Ministry of Health, Labour and Welfare [14].

Statistical analysis

We evaluated the temporal trends in the number of EF cases, number of fracture fixations, and EF usage ratio using Cochrane-Armitage trend test for nationwide population. We also examined the data stratified for each prefecture. The regional difference of EF usage ratio was described by the histogram and geographical plot. We analyzed the association of the EF usage ratio in each prefecture over a five-year period, and also related factors (population, regional classification, the number of tertiary emergency and critical care centers per one million people) using multiple regression analyses. Results were considered statistically significant at p < 0.05. Statistical analysis was conducted using R version 4.4.1. Geographical plot was generated using Python.

## Results

**Figure 1:**
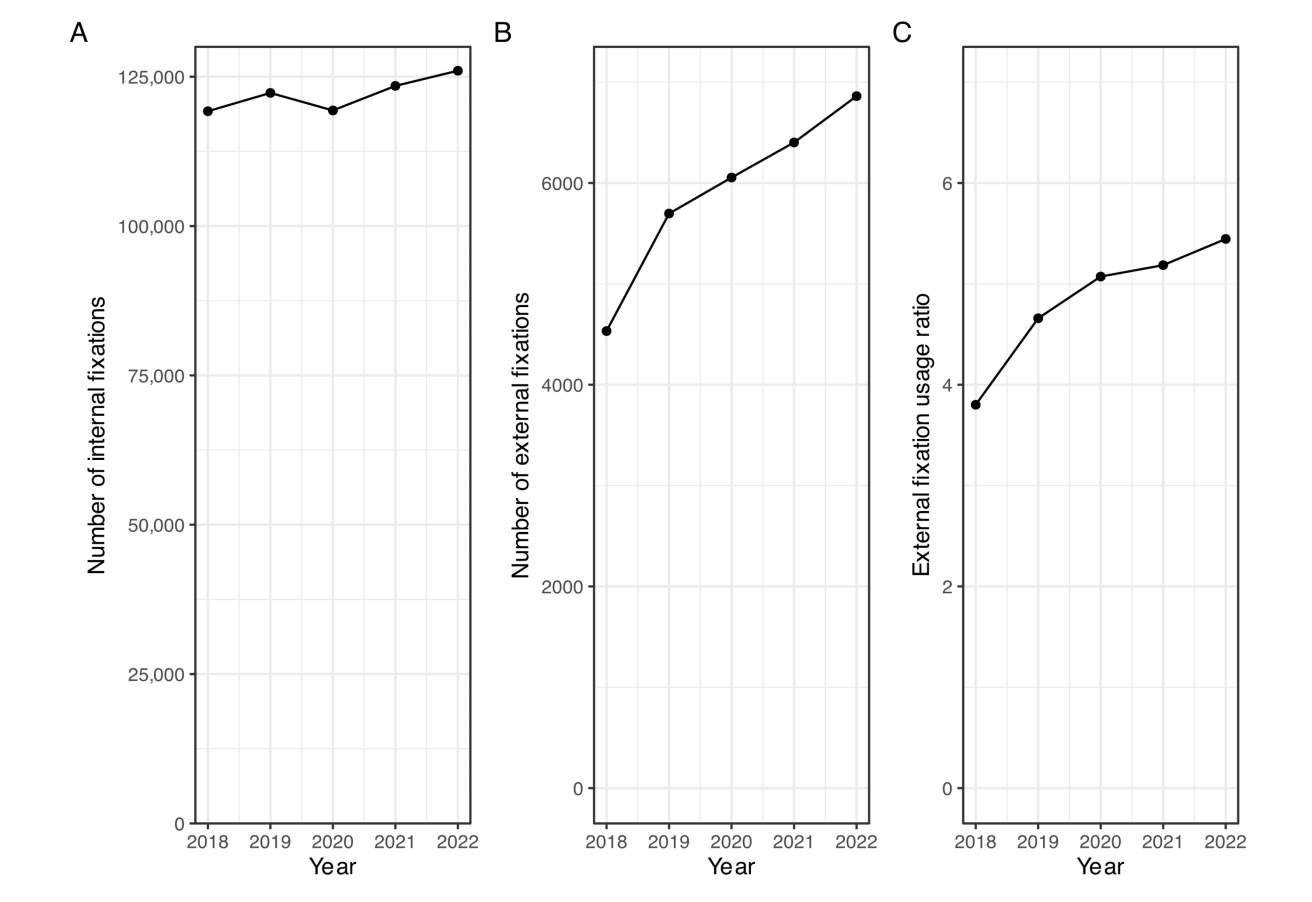
Temporal trend of the number of internal fixations, EFs, and EF usage ratio A. Number of internal fixations from 2018 to 2022; B. Number of EFs from 2018 to 2022; C. EF usage ratio from 2018 to 2022 EF: External fixation

During the five-year period, we included total numbers of 610,326 internal fixations and 29,546 primary EFs from open NDB data. The EF usage ratio was 4.8% (29,546/610,326). The trend of the numbers of internal fixations, primary EFs and the EF usage ratio are described as Figure 1. While there is no increase in the number of internal fixations, there is a continuous increase in the number of EFs from 4,532 cases in 2018 to 6,861 cases in 2022. The EF usage ratio also increased from 4,532 out of 119,223 cases (3.8%) in 2018 to 6,861 out of 126,000 cases (5.4%) in 2022 (p < 0.01). The trend of the number of internal fixations, primary EFs, EF usage ratio in each prefecture is described in Appendix 1. Among 47 prefectures, 12 showed the increase trend of EF usage ratio over five-year period (i.e., Aomori, Iwate, Yamagata, Tochigi, Saitama, Chiba, Tokyo, Kyoto, Osaka, Okayama, Kagoshima, Okinawa) and one prefecture showed the decrease trend of EF usage ratio (Toyama).

**Figure 2:**
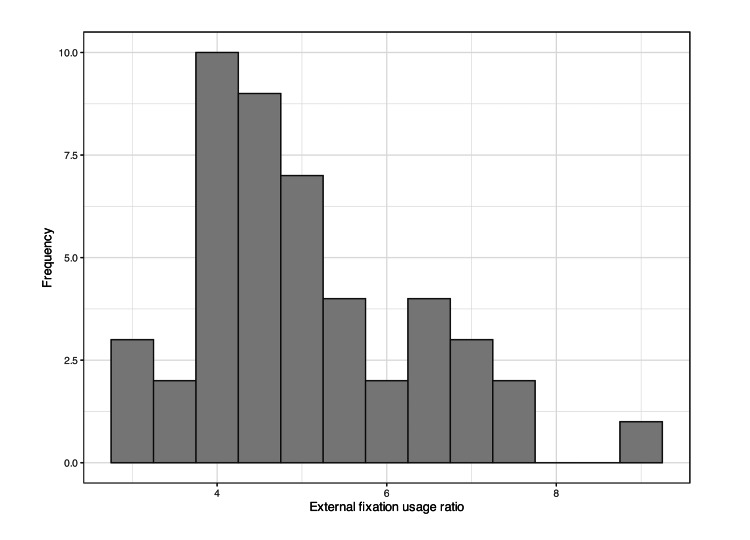
The distribution of EF usage ratio among prefectures EF: External fixation

**Figure 3:**
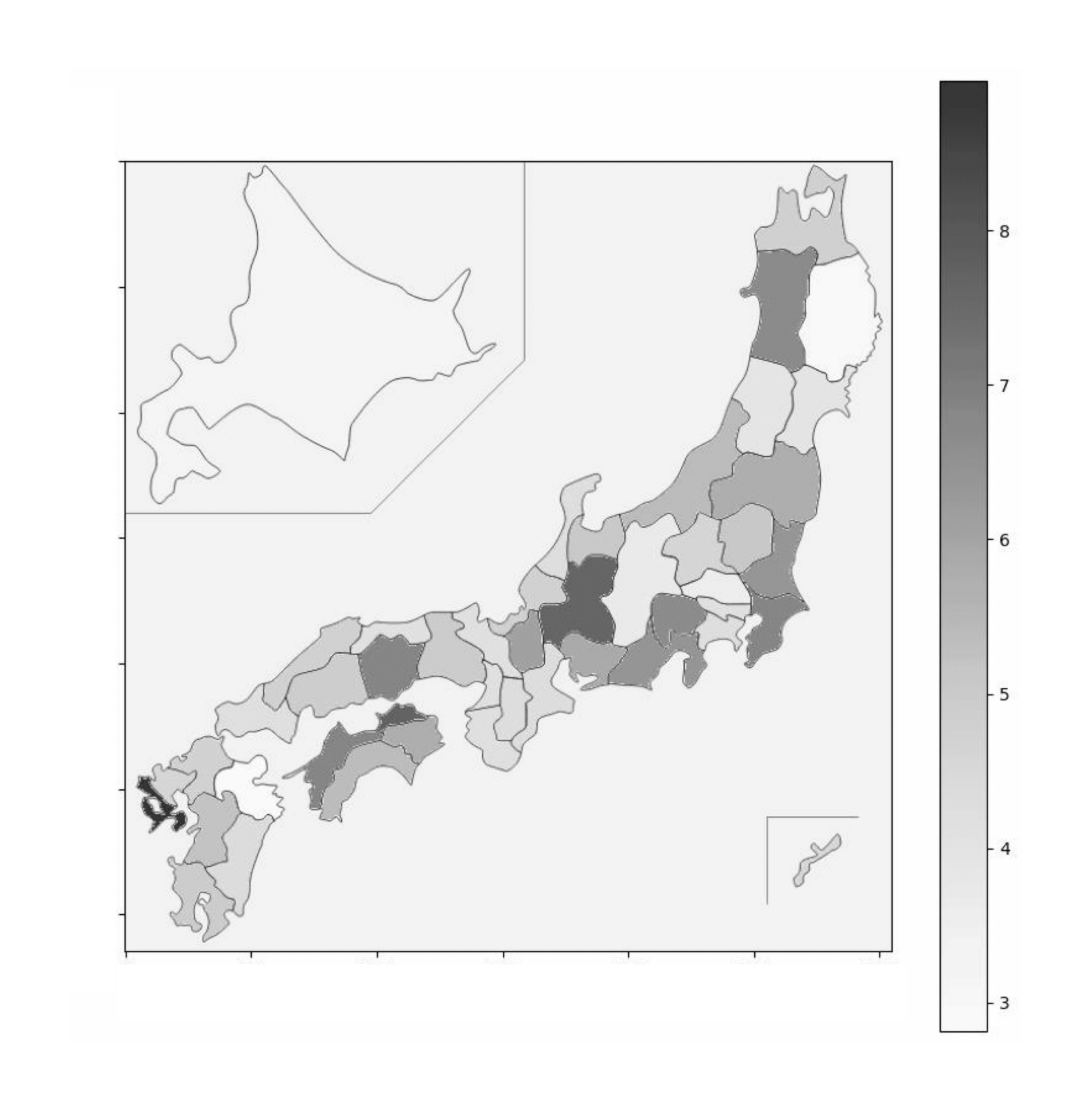
Regional difference of EF usage ratio in each prefecture over five-year period EF: External fixation

The regional difference of EF usage ratio over a five-year period is described in the figures below (Figures 2,3). The EF usage ratio among each prefecture is slightly skewed to the left, and the median percentage is 4.7% (Figure 2). The EF usage ratio is the lowest in Oita Prefecture at 2.8% (228/8,103) and highest in Nagasaki Prefecture at 9.0% (814/9,075), representing a threefold difference (Figure 3).

Table 1 shows the association between EF usage ratio and potential related factors. We could not identify the related factors that would be associated to the EF usage ratio among each prefecture.

**Table 1:** Association between potential relevant factors and the increase of EF usage ratio EF: External fixation

	Beta	95% CI	p-value
Intercept	5.4	3.7, 7.0	< 0.001
Population	0	0.00, 0.00	0.2
Number of tertiary care centers	-0.22	-0.68, 0.25	0.4
Region	0.1	-0.09, 0.30	0.3

## Discussion

This study analyzed the use of EFs over a five-year period using the open NDB database, revealing a gradual increase in both the number of EFs performed and the EF usage ratios, which rose from 3.8% (4,532/119,223) in 2018 to 5.4% (6,861/12,600) in 2022. Although the overall number of internal fixations remained stable, the steady rise in EF usage suggests a growing preference for this method. The EF usage ratio varied by region, with a threefold difference between the lowest (2.8% in Oita) and highest (9.0% in Nagasaki). Despite these regional variations, the study could not identify specific factors that might explain the differences in EF usage across prefectures.

Potential mechanism of our findings

Financial incentives, particularly those linked to reimbursement changes, may be a contributing factor to the increase in EF usage in fracture care. Several studies have explored the impact of financial incentives on changing clinical practice, including smoking cessation programs, quality of care in primary practice in the UK, and diabetes care in Italy [15-17]. A systematic review of 32 studies found that financial incentives might influence changes in healthcare professionals' practices [18]. Additionally, in the Japanese healthcare system, financial incentives on early mobilization and rehabilitation in intensive care units have led to shorter hospital stays and improved patients outcome [19].

Before 2018, primary EF was reimbursed as part of debridement surgery at a rate of 3,090 points ($206 ($1 = 150 Yen)). After 2018, a specific reimbursement fee for primary EF (K046-3, code 150395810) was implemented at 34,000 points ($2,266). In the Japanese healthcare system, orthopedic implant costs are reimbursed through medical insurance, separate from surgical fees. However, EF devices, which are not retained within patients, do not qualify as implants and thus are not eligible for reimbursement, creating a financial burden for hospitals. To offset this, reimbursement for the surgery itself was increased to cover the costs of EF. These fees are determined by the complexity of the procedure, the duration, and the associated medical expenses, including personnel and facility use. This adjustment reduces barriers to utilizing primary EF for damage control surgeries. Although the number of EFs before 2018 is unknown, this change may have acted as a financial incentive, contributing to the increase in both the number and usage ratio of EF.

Another potential reason for the increased EF usage ratio is the awareness toward damage control in orthopedic trauma care. In the field of distal radius fracture care, a guideline change in Denmark and the result of large randomized controlled trial in the UK have been reported to change the clinical practice [20,21]. The recent publication of US guidelines and related studies, which emphasize the importance of damage control, may have increased awareness within orthopedic trauma [1-3]. This awareness may be attributed to an increase of the number of surgeons/facilities that prioritize damage control in orthopedic trauma care in Japan.

We could not find the significant factor related to the regional difference in the usage ratio of EF. However, a survey on lateral ankle sprain management in Japan reveals that clinical practices vary among orthopedic surgeons, even within the same prefecture, and suggests that one reason for this variation is a lack of knowledge about the evidence [22]. Another reason is the trauma care system in Japan. Unlike the US or Europe, which have specific orthopedic trauma care rooms, some Japanese facilities lack sufficient operating rooms or staff dedicated to emergent or urgent orthopedic care, leaving no choice but to perform damage control using skeletal traction or splinting. Alternatively, some surgeons or facilities opt for definitive internal fixation in a single stage rather than EF for open or comminuted fractures [23]. Variation in the trauma education and trauma care system may have affected the regional variation of EF usage ratio.

Limitations

This study represents the first to demonstrate the trends and regional variations in orthopedic surgeries recently incorporated into the health insurance reimbursement system. A major strength of this study is the use of the NDB, a comprehensive database covering all electronic claims within Japan's national health insurance system.

However, this study has several limitations. First, since the NDB system does not contain the information covered by industrial incident compensation insurance, mandatory vehicle liability insurance, or compensation for damage, it inherently excludes certain fractures resulting from traffic and industrial accidents, which are typically more severe than those from other causes [24]. As a result, the ratio of EF use among fractures may be underestimated. Second, the NDB data was collected for reimbursement and not for collecting data for specific research and has not been validated [24]. Therefore, it lacks the information on fracture severity and specific anatomical locations (e.g., distal, diaphyseal, proximal), making it impossible to determine the precise indication for primary EF in each case [24]. However, over the course of five years, most of the regions with low ratios have consistently remained low, while those with high ratios have consistently remained high with the gradual increase within the region. This indicates that the estimated ratios in this study reflect real differences in treatment policies across the regions. Third, open NDB data is aggregate data. It does not contain any individual data and anatomical location of the EF is not included in the open data. Therefore, the association between primary EF placement and patient outcomes cannot be clarified. Furthermore, determining the anatomical characteristics of the fracture and assessing surgeon and center preferences as factors influencing the indication for EF remains unfeasible. To address these limitations, similar studies could be conducted using other databases, such as trauma registries. Fourth, since specific reimbursement for primary EF began in 2018, no data is available on EF usage before that year, making it impossible to determine the actual effect of the reimbursement. Lastly, since this study is centered on the Japanese system, its findings cannot be generalized to contexts in other countries. However, these findings may be applicable to other areas within orthopedics under Japanese medical insurance.

Clinical and policy implication of this study

Primary EF is typically considered as a simple, temporary treatment method, often performed by early career surgeons. However, several pitfalls exist, including safe zone pin placement, optimal frame construction, and pre-surgical planning for converting EF to internal fixation [2]. With the increasing use of primary EF, there is a need to raise awareness of these challenges and to enhance education for early career surgeons.

As previously mentioned, financial reimbursement has been reported to influence clinical practice in the field of rehabilitation within the Japanese healthcare system [19]. This effect has not been analyzed in the field of orthopedic surgery. Our study suggests a potential influence of financial reimbursement on orthopedic trauma care; however, the lack of pre-reimbursement data on primary EF limits our findings. Future research should employ more robust methodologies such as interrupted time series analysis to clarify the impact of financial reimbursement on orthopedic clinical practice.

## Conclusions

This study identified a gradual increase in EF usage over five years, rising from 3.8% in 2018 to 5.4% in 2022, while the use of internal fixations remained stable. EF utilization varied across regions, ranging from 2.8% in Oita Prefecture to 9.0% in Nagasaki Prefecture, though the specific factors driving these regional differences remain unidentified. Future research is required to reveal the underlying causes of these disparities, such as variations in local clinical practices, resource availability, and patient demographics, to facilitate the standardization of orthopedic trauma care throughout Japan.

## Appendices

Appendix 1

**Table 2:** Trend of number of EFs and internal fixations, and EF usage ratio among each prefecture *P-value by Cochrane-Armitage test EF: External fixation

Prefecture	Number of EFs					Number of internal fixations					EF usage ratio					P-value*
	2018	2019	2020	2021	2022	2018	2019	2020	2021	2022	2018	2019	2020	2021	2022	
Hokkaido	142	196	185	197	220	5,865	6,145	5,833	6,148	6,242	2.4	3.2	3.2	3.2	3.5	0.06
Aomori	27	28	53	50	81	998	938	1,024	1,062	950	2.7	3.0	5.2	4.7	8.5	0.03
Iwate	26	31	47	47	45	1,348	1,276	1,399	1,335	1,272	1.9	2.4	3.4	3.5	3.5	0.02
Miyagi	67	62	64	84	81	1,754	1,663	1,979	1,983	1,736	3.8	3.7	3.2	4.2	4.7	0.24
Akita	48	34	84	95	89	984	902	1,101	1,195	1,072	4.9	3.8	7.6	7.9	8.3	0.07
Yamagata	25	30	35	49	50	939	861	1,002	1,037	1,008	2.7	3.5	3.5	4.7	5.0	0.01
Fukushima	100	102	120	80	109	1,770	1,658	1,689	1,907	1,867	5.6	6.2	7.1	4.2	5.8	0.70
Ibaraki	99	165	147	158	166	2,149	2,297	2,314	2,294	2,513	4.6	7.2	6.4	6.9	6.6	0.31
Tochigi	52	77	98	104	93	1,664	1,638	1,745	1,735	1,591	3.1	4.7	5.6	6.0	5.8	0.04
Gunma	65	71	81	84	66	1,666	1,601	1,579	1,569	1,536	3.9	4.4	5.1	5.4	4.3	0.45
Saitama	139	202	184	234	292	5,509	6,068	5,612	5,878	6,275	2.5	3.3	3.3	4.0	4.7	0.01
Chiba	320	357	352	395	458	5,276	5,505	5,300	5,643	5,763	6.1	6.5	6.6	7.0	7.9	0.01
Tokyo	360	443	469	491	605	11,742	11,946	10,445	11,560	11,749	3.1	3.7	4.5	4.2	5.1	0.02
Kanagawa	229	297	366	399	399	7,594	7,985	7,405	8,396	8,748	3.0	3.7	4.9	4.8	4.6	0.10
Niigata	64	112	147	131	146	2,027	2,149	2,391	2,209	2,366	3.2	5.2	6.1	5.9	6.2	0.08
Toyama	65	56	54	54	54	1,125	1,069	1,094	1,197	1,172	5.8	5.2	4.9	4.5	4.6	0.02
Ishikawa	45	54	57	41	62	1,085	1,249	1,241	1,239	1,283	4.1	4.3	4.6	3.3	4.8	0.88
Fukui	39	39	30	36	32	688	750	743	753	792	5.7	5.2	4.0	4.8	4.0	0.10
Yamanashi	46	65	63	59	48	860	875	813	811	860	5.3	7.4	7.7	7.3	5.6	0.94
Nagano	46	61	87	91	101	1,908	2,003	1,978	2,375	2,293	2.4	3.0	4.4	3.8	4.4	0.06
Gifu	78	108	132	118	113	1,376	1,367	1,385	1,595	1,469	5.7	7.9	9.5	7.4	7.7	0.50
Shizuoka	185	169	202	216	213	2,950	3,039	3,084	3,112	3,212	6.3	5.6	6.5	6.9	6.6	0.25
Aichi	289	384	355	348	334	5,633	5,762	5,789	5,969	6,042	5.1	6.7	6.1	5.8	5.5	0.99
Mie	51	67	89	107	77	1,744	1,775	1,862	1,820	1907	2.9	3.8	4.8	5.9	4.0	0.27
Shiga	41	69	80	71	102	1,064	1,206	1,151	1,171	1379	3.9	5.7	7.0	6.1	7.4	0.06
Kyoto	91	88	102	130	140	2,608	2,708	2,452	2,753	2,872	3.5	3.2	4.2	4.7	4.9	0.02
Osaka	304	373	392	425	455	9,404	9,426	9,158	9,264	9,759	3.2	4.0	4.3	4.6	4.7	0.01
Hyogo	185	292	294	302	328	5,585	5,777	5,502	5,702	5,910	3.3	5.1	5.3	5.3	5.5	0.09
Nara	71	65	68	97	82	1,846	1,892	1,743	1,677	1,765	3.8	3.4	3.9	5.8	4.6	0.21
Wakayama	32	35	56	44	75	1,147	1,160	1,206	1,124	1,143	2.8	3.0	4.6	3.9	6.6	0.05
Tottori	27	21	27	30	27	583	681	684	681	613	4.6	3.1	3.9	4.4	4.4	0.72
Shimane	26	35	40	38	35	764	752	716	734	705	3.4	4.7	5.6	5.2	5.0	0.19
Okayama	105	134	140	185	192	2,290	2,245	2,161	2,212	2,161	4.6	6.0	6.5	8.4	8.9	< 0.01
Hiroshima	143	152	143	152	166	3,049	3,057	3,043	3,126	3,160	4.7	5.0	4.7	4.9	5.3	0.20
Yamaguchi	47	55	68	69	61	1,461	1,391	1,497	1,435	1,417	3.2	4.0	4.5	4.8	4.3	0.12
Tokushima	40	45	32	30	47	666	659	706	701	644	6.0	6.8	4.5	4.3	7.3	0.99
Kagawa	80	82	93	105	88	1,183	1,229	1,193	1,110	1,104	6.8	6.7	7.8	9.5	8.0	0.16
Ehime	66	126	121	122	112	1,547	1,637	1,579	1,581	1,643	4.3	7.7	7.7	7.7	6.8	0.34
Kochi	37	56	37	44	18	709	770	768	680	661	5.2	7.3	4.8	6.5	2.7	0.36
Fukuoka	235	326	286	265	290	5,745	6,089	6,131	5,840	6,021	4.1	5.4	4.7	4.5	4.8	0.72
Saga	49	54	62	46	64	1,176	1,233	1,193	1,195	1,217	4.2	4.4	5.2	3.8	5.3	0.49
Nagasaki	124	155	135	196	204	1,874	1,824	1,836	1,732	1,809	6.6	8.5	7.4	11.3	11.3	0.05
Kumamoto	75	105	107	109	117	2,057	1,954	1,932	1,917	1,973	3.6	5.4	5.5	5.7	5.9	0.07
Oita	31	43	56	47	51	1,565	1,681	1,551	1,606	1,700	2.0	2.6	3.6	2.9	3.0	0.25
Miyazaki	31	47	52	52	59	1,044	1,076	1,085	1,171	1,180	3.0	4.4	4.8	4.4	5.0	0.09
Kagoshima	49	86	97	104	113	1,716	1,764	1,822	1,834	1,928	2.9	4.9	5.3	5.7	5.9	0.04
Okinawa	36	43	65	71	101	1,375	1,477	1,333	1,340	1,438	2.6	2.9	4.9	5.3	7.0	< 0.01
Total	4,532	5,697	6,054	6,402	6,861	119,223	122,286	119,353	123,464	126,000	3.8	4.7	5.1	5.2	5.4	0.02
